# Customized 3D-printed component-preserving reconstruction for femoral neck fracture revision in a long-term survivor of pediatric osteosarcoma: a case report

**DOI:** 10.3389/fsurg.2026.1819982

**Published:** 2026-05-26

**Authors:** Zhiming Yu, Qi Sun, Xinyu Nan, Run Liu, Liuxin Yan, Hui Wang, Yang Lu, Yaheng Zhao, Gaocen Li, Lufeng Lin, Huiyang Jia, Changcheng Liu

**Affiliations:** 1 Hip Joint Center, The First Hospital of Hebei Medical University, Shijiazhuang, China; 2 School of Nursing, Hebei Medical University, Shijiazhuang, China

**Keywords:** 3D printing, case report, component-preserving reconstruction, femoral neck fracture, osteosarcoma

## Abstract

**Background:**

Late proximal femoral fractures in long-term survivors with childhood tumor endoprostheses are rare and technically challenging. When the proximal femur lacks a usable medullary canal but the distal tumor prosthesis remains stable, reconstruction is difficult and total femoral replacement may be excessively invasive.

**Case presentation:**

A 28-year-old man with a history of distal femoral osteosarcoma treated in childhood with knee tumor prosthesis replacement presented after a fall with severe right hip pain and inability to bear weight. Imaging confirmed a displaced femoral neck fracture, while the distal prosthesis remained well fixed. To preserve the stable distal component, we performed a component-preserving reconstruction using a customized monoblock stem with a socket-type prosthesis-to-prosthesis coupling design. The construct was stabilized with vancomycin-loaded PMMA cement, locking screws, and a lateral anti-rotation plate, and a dual-mobility head was implanted. Postoperative radiographs showed satisfactory alignment and positioning. Limb-length discrepancy improved from approximately 20 cm to 15 cm. At 3 months, the Harris Hip Score was 63; at approximately 6 months, it improved to 81, with continued functional recovery.

**Conclusion:**

This case suggests that customized component-preserving reconstruction may be a feasible alternative to total femoral replacement in selected patients with complex proximal femoral defects and retained stable distal tumor prostheses. Longer follow-up is needed to determine durability and late complications.

## 1 Introduction

Osteosarcoma is one of the most common primary malignant bone tumors in children and adolescents, accounting for approximately 6% of all malignancies in this population. It typically arises in the metaphyseal regions of long bones, most commonly in the femur (1, 2). With advances in chemotherapy and limb reconstruction techniques, limb-salvage surgery has become the standard treatment, significantly improving long-term survival (3). However, in pediatric patients, damage to the epiphyseal plate often results in impaired skeletal development. As growth continues, this may lead to limb length discrepancy, osteoporosis, and periprosthetic fractures (4–6).

Periprosthetic fracture is one of the most common yet challenging complications encountered during long-term follow-up after tumor prosthesis implantation (7). Such patients often exhibit marked bone loss, insufficient load-bearing bone stock, and varying degrees of soft tissue damage, all of which increase the complexity and risk of revision surgery (8). Traditional total femoral replacement (TFR) involves extensive surgical trauma and delayed functional recovery, significantly affecting the quality of life in younger patients. In recent years, 3D printing has demonstrated unique advantages in the design of personalized prostheses and precise anatomical reconstruction (9).

This report describes a rare case of periprosthetic fracture (femoral neck fracture following knee tumor prosthesis replacement). The patient, who underwent distal femoral osteosarcoma resection and total knee tumor prosthesis replacement at the age of 8 in 2005, later developed proximal prosthesis migration and greater trochanteric perforation due to mid-femoral bone loss, which required revision surgery in 2018. Recently, the patient sustained a fall that caused a femoral neck fracture on the prosthetic side. Because the femur lacked a normal medullary canal, standard femoral stems were unsuitable for total hip arthroplasty (THA). After comprehensive preoperative evaluation and design, we performed component-preserving reconstruction using a customized monoblock stem combined with a dual-mobility head, achieving successful functional recovery. Here, we propose a component-preserving reconstruction strategy based on a socket-type prosthesis-to-prosthesis coupling system to address a complex femoral defect with a retained distal implant. The innovation of this case lies in three aspects: strategically, it avoided total femoral replacement by preserving a stable distal implant; structurally, it used a socket-type prosthesis-to-prosthesis coupling design; and mechanically, it employed hybrid fixation with vancomycin-loaded PMMA cement, locking screws, and a lateral anti-rotation plate. This report aims to demonstrate the technical feasibility and early clinical outcome of this component-preserving reconstruction approach.

## 2 Case presentation

### 2.1 Patient information and clinical findings

**Figure 1:**
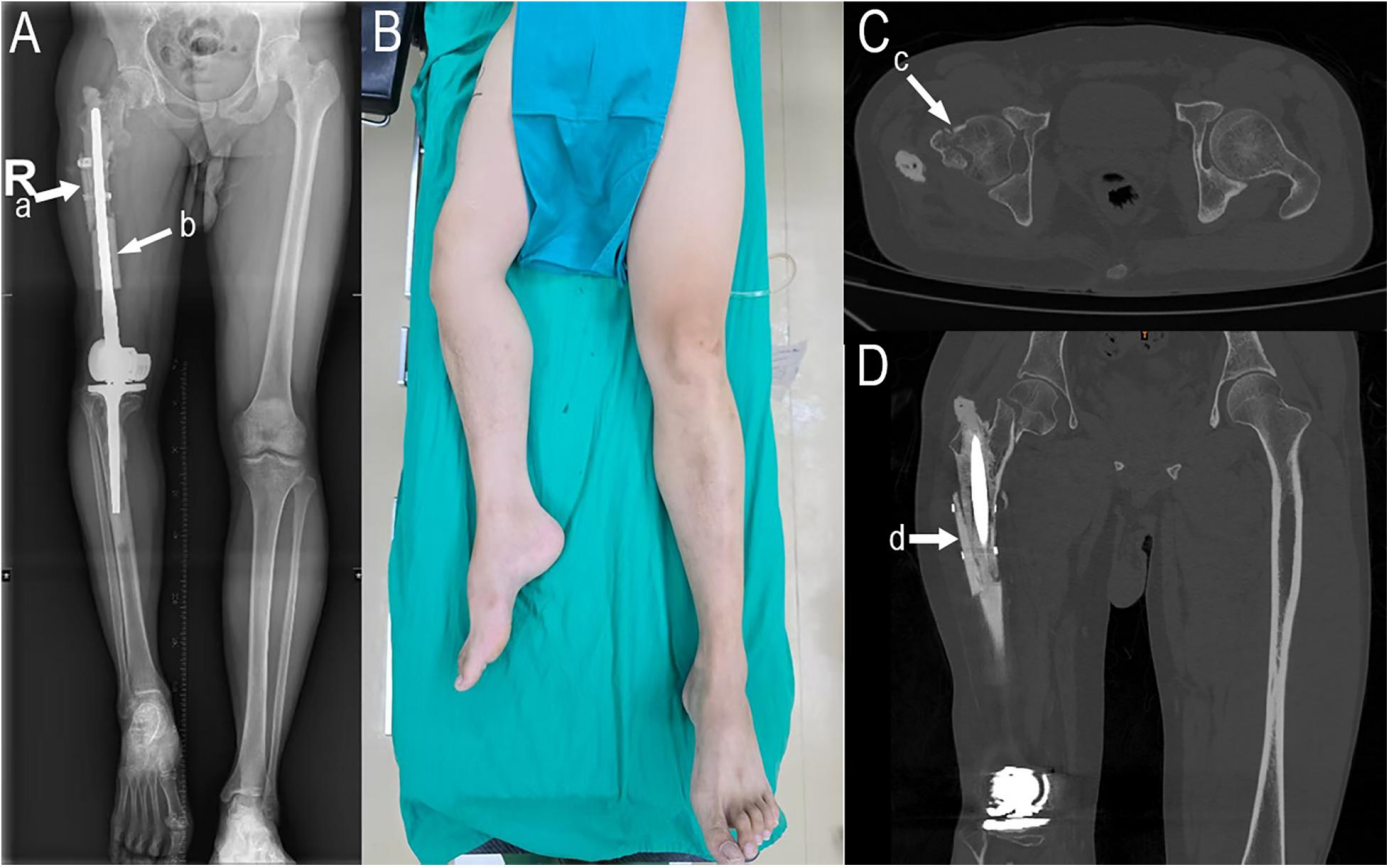
Preoperative radiographic and computed tomography findings. **(A)** Full-length anteroposterior radiograph of both lower limbs shows the retained femoral tumor endoprosthesis and prior proximal femoral reinforcement; the cerclage wire at the previous fracture site (a) and the homogeneous “artificial bone plate” construct (b) are indicated. **(B)** Preoperative operating-room photograph demonstrates marked right lower-limb muscle atrophy with limb shortening. **(C)** Axial CT image demonstrates the femoral neck fracture (c). **(D)** Coronal CT reconstruction demonstrates the corresponding fracture/reinforcement region (d), consistent with the cerclage-wire location in panel **(A)** (a) CT = computed tomography.

The patient was a 28-year-old man who was diagnosed with osteosarcoma of the right distal femur in 2005 at age 8 and underwent tumor resection with total knee tumor prosthesis replacement. He had no known additional comorbidities or relevant chronic medical conditions. Postoperative function of the prosthesis was satisfactory, with no local recurrence or distant metastasis. In 2018, owing to inadequate bony support in the mid-femoral segment, proximal migration of the knee tumor prosthesis led to greater trochanteric perforation and a palpable prominence beneath the skin; revision surgery was therefore performed. During revision surgery, an artificial bone plate was implanted at the mid-femoral segment for reinforcement, the proximal femoral canal was filled with polymethylmethacrylate (PMMA) bone cement for fixation, and cerclage wiring was applied across the fracture site to enhance overall stability. Recently, the patient sustained a fall and presented with right hip pain, restricted mobility, and inability to bear weight (Figure 1). Physical examination revealed well-healed surgical scars from the right thigh to the lower leg, marked right lower-limb muscle atrophy, and an obvious limb-length discrepancy, with the right leg shortened by approximately 20 cm compared with the left. The right hip range of motion was markedly limited due to pain, whereas the right knee and contralateral hip and knee ranges of motion were preserved.

After discussion with the patient and multidisciplinary consultation, two surgical options were considered: total femoral replacement or component-preserving reconstruction using a customized monoblock stem. Given that the distal prosthesis appeared well fixed and the patient preferred maximal component preservation, proximal reconstruction with retention of the distal tumor prosthesis was selected.

### 2.2 Prosthesis design

**Figure 2:**
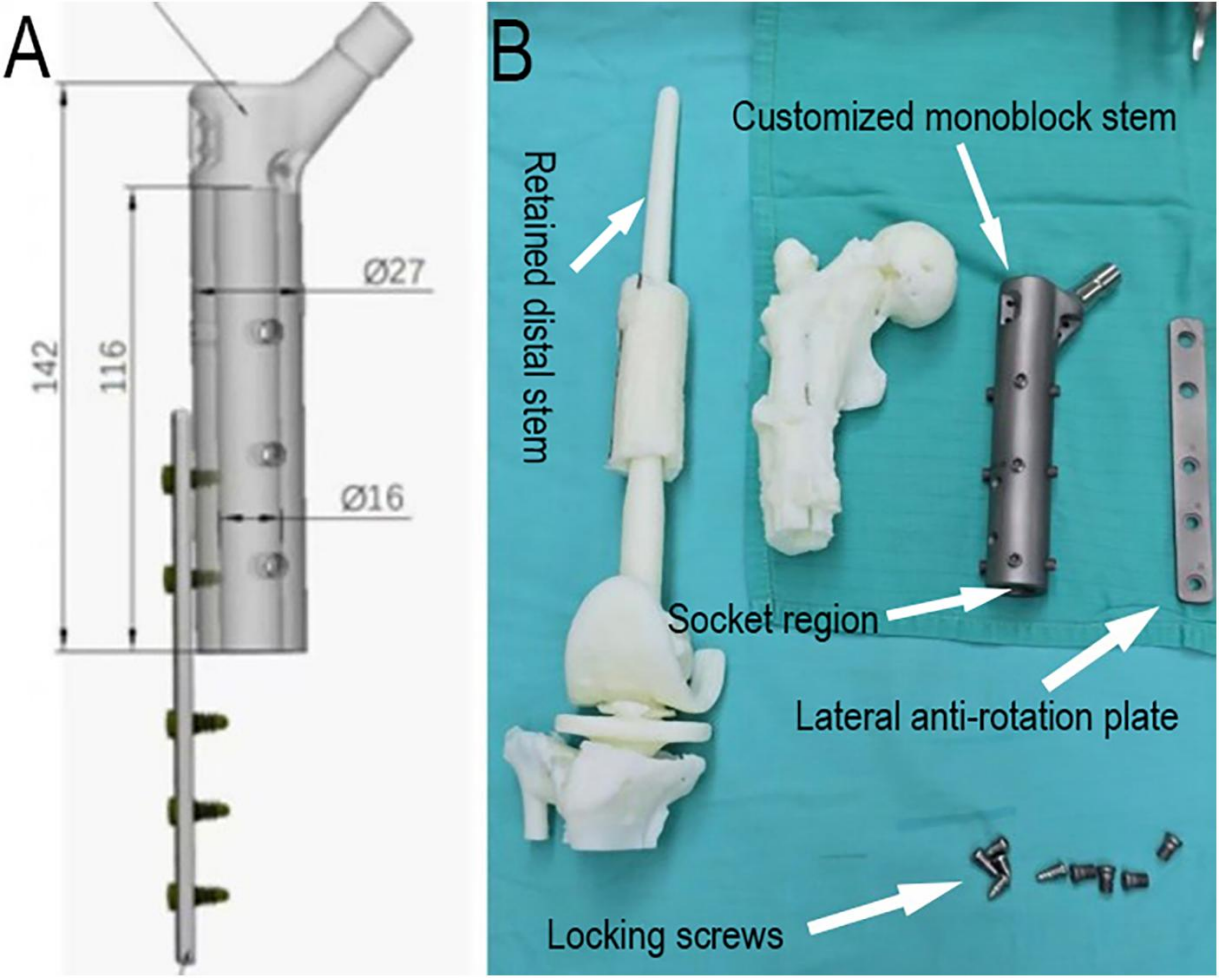
Design and conceptual structure of the component-preserving reconstruction. **(A)** Computer-aided design of the customized monoblock stem with a distal hollow socket for prosthesis-to-prosthesis coupling with the retained distal tumor prosthesis; key dimensions are shown. **(B)** Schematic illustration of the prosthesis-to-prosthesis coupling interface, showing the customized monoblock stem, socket region, retained distal stem, locking screws, and lateral anti-rotation plate.

Preoperative CT and radiographs were obtained for three-dimensional reconstruction to measure the geometry of the residual femoral segment and the retained distal prosthesis. To preserve the original distal knee tumor prosthesis and reconstruct proximal and mid-femoral defects, 3D printing was used to create solid models of the femoral remnant and the proposed customized prosthesis for intraoperative simulation. The prosthesis design included a customized monoblock stem, multiple locking screws, and an anti-rotation plate (Figure 2). The femoral stem was designed according to the morphology of the residual segment and the anticipated postoperative limb length, with an overall length of 142 mm, external diameter of 27 mm, internal canal length of 116 mm, and internal diameter of 16 mm. The proximal interface was compatible with a dual-mobility femoral head, while the distal end was designed as a hollow socket to accept the retained distal prosthesis stem. Vancomycin-loaded PMMA cement was used to bond the customized monoblock stem to the residual distal prosthesis, with additional fixation by multiple locking screws and a lateral anti-rotation plate to resist torsion, shear, and axial displacement. Vancomycin-loaded PMMA was used prophylactically given the high-risk revision setting with multiple prior operations and extensive scar tissue.

### 2.3 Surgical procedure

**Figure 3:**
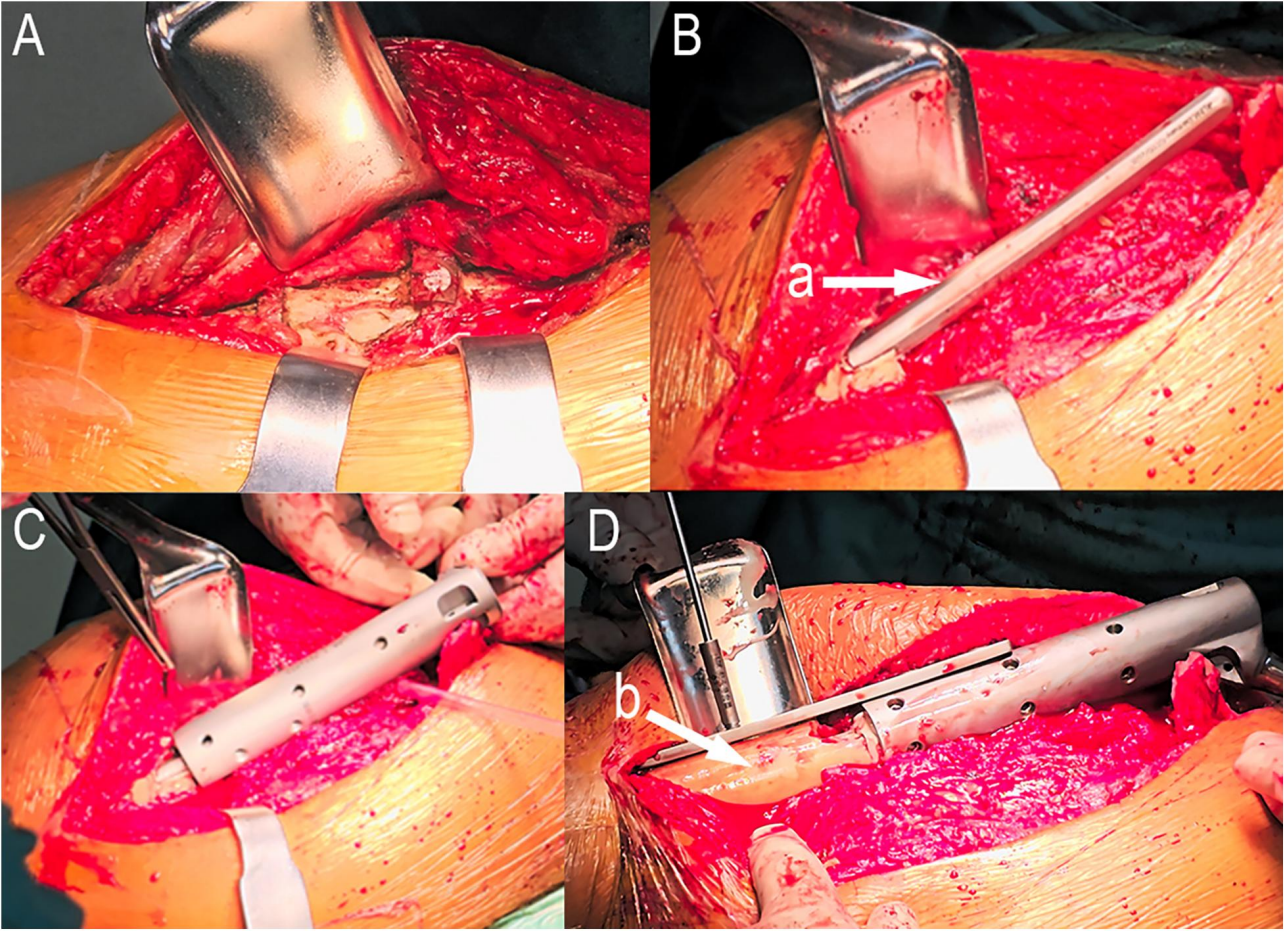
Intraoperative views of the previous mid-femoral fracture/reinforcement region, the retained endoprosthesis, and coupling of the customized implant. The exposed area corresponds to region d in Figure 1D (left = foot side; right = head side). **(A)** Exposure of the mid-femoral fracture/reinforcement region. **(B)** The retained femoral tumor knee endoprosthesis (a). **(C)** Coupling of the socket region of the customized monoblock stem with the retained distal prosthesis. **(D)** Final positioning of the customized construct; the homogeneous mid-femoral “artificial bone plate” structure (b, corresponding to Figure 1A) is shown.

Under general anesthesia, the patient was placed in the right lateral decubitus position. Through the original lateral incision on the right thigh, approximately 40 cm in length, the previous surgical approach was reopened. The skin and subcutaneous tissue were dissected layer by layer, revealing significant muscle atrophy and dense scar adhesions. The iliotibial band and vastus lateralis were incised, exposing the fractured proximal femoral bone segment, which had been stabilized with metallic cerclage bands and was surrounded by fibrotic tissue (Figure 3). The cerclage bands and associated fibrotic tissue were removed as needed to facilitate exposure and reconstruction.

From the lateral aspect, the greater trochanter was split to gain exposure while preserving the insertions of the short external rotators and the gluteus medius. The acetabulum was exposed, and a femoral neck fracture was identified. The fractured femoral head was extracted, and the joint cavity was thoroughly debrided and protected with gauze packing. For infection prophylaxis, cefazolin sodium was administered intravenously 30 min before incision. Intraoperatively, the wound, surrounding soft tissues, and residual bone fragments were irrigated thoroughly with copious normal saline before and after prosthesis implantation. No microbiological assessment was performed because there was no clinical suspicion of active infection. After complete debridement and copious irrigation of the surgical field, the fit of the femoral remnant was assessed using a 3D-printed model, which demonstrated excellent congruence.

The customized monoblock stem was cemented to the distal prosthesis remnant using vancomycin-loaded PMMA bone cement. The anteversion and rotational alignment of the stem were meticulously adjusted, and multiple locking screws were inserted for fixation. A lateral anti-rotation plate was then installed and secured to the customized monoblock stem and the residual proximal femoral segment to enhance resistance to torsional, shear, and axial displacement (Figure 3). A dual-mobility femoral head was trialed and implanted, showing satisfactory stability and appropriate version. The residual trochanteric bone fragments with muscular attachments were repositioned and secured using two titanium cables in a figure-of-eight configuration through the predesigned holes of the prosthesis. After confirming appropriate muscle tension and construct stability, the operative field was irrigated thoroughly, hemostasis was achieved, a negative-pressure drainage tube was inserted, and the incision was closed in layers with compressive dressing applied.

### 2.4 Follow-up and outcomes

**Figure 4:**
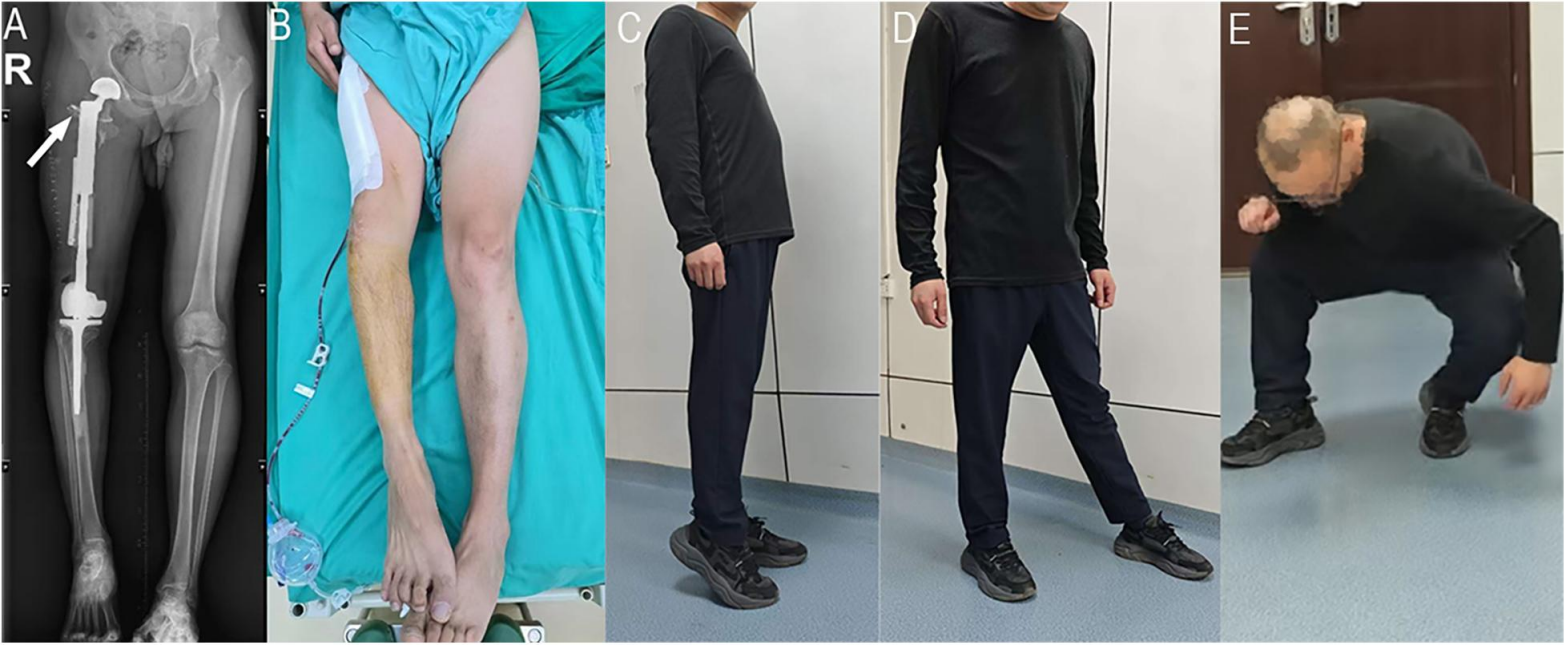
Postoperative outcomes. **(A)** Full-length anteroposterior radiograph showing satisfactory position of the customized construct; the arrow indicates the titanium-cable–reconstructed soft-tissue attachment site. **(B)** Supine photograph of the lower limbs. **(C)** Standing with the contralateral limb as the primary weight-bearing limb. **(D)** Standing with the affected limb as the primary weight-bearing limb. **(E)** Squatting posture.

On postoperative day 4, the negative-pressure drainage tube was removed, with daily drainage volumes of 260 mL, 250 mL, and 100 mL during the first three postoperative days. No major postoperative complications were observed. A transient elevation in C-reactive protein was noted on routine postoperative laboratory testing; however, no corresponding clinical evidence of infection or other clinically significant complication was identified. Immediate postoperative anteroposterior and lateral radiographs demonstrated satisfactory alignment and positioning of the customized construct, with no obvious early mechanical instability on available imaging (Figure 4). The limb-length discrepancy improved from approximately 20 cm preoperatively to 15 cm postoperatively. Further correction of limb-length discrepancy was not pursued because of concerns regarding excessive soft-tissue tension and potential neurovascular compromise in the setting of long-standing deformity and multiple prior surgeries. During the first 5 postoperative days, the patient remained on bed rest and performed lower-limb flexion-extension exercises, ankle pump exercises, and pneumatic compression therapy for thromboprophylaxis. On postoperative day 5, he began simple standing training under the guidance of a rehabilitation therapist. Hip flexion beyond 90°, adduction beyond the midline, and excessive internal rotation on the operated side were avoided. The patient was discharged 1 week after surgery and continued walking exercises at home with family assistance. To address the residual limb-length discrepancy, a shoe lift or platform shoe was recommended, although the patient did not adopt this recommendation.

At the 3-month follow-up, the patient achieved independent ambulation over short distances, although a noticeable limp persisted. He reported occasional mild pulling discomfort in the gluteal region during hip motion, which gradually improved with gentle activity and routine rehabilitation exercises. Because of the retained distal knee tumor prosthesis, squatting remained limited; the patient could not perform a full squat and was mainly able to sit down onto a chair or stool. The Harris Hip Score at this follow-up was 63.

At approximately 6 months postoperatively, the patient reported further improvement in walking capacity, subjectively exceeding the pre-fracture level, and he was able to perform daily activities without major limitations. Nonetheless, squatting remained restricted and was predominantly achieved by relying on the contralateral (uninjured) limb for power and support. The Harris Hip Score improved to 81 at this follow-up. Clinical photographs demonstrating early functional recovery are provided in Figure 4.

### 2.5 Patient perspective

After the injury, the patient reported severe pain, inability to bear weight, and marked limitation in mobility. During the preoperative discussion, he expressed a clear preference for preserving the stable distal tumor prosthesis if possible and agreed to the component-preserving reconstruction after discussion of the potential risks and benefits in comparison with total femoral replacement. During follow-up, he reported improved walking ability and daily function compared with the preoperative condition. Although residual limb-length discrepancy, limp, and limited squatting persisted, he reported acceptance of the overall outcome and valued preservation of the distal prosthesis.

## 3 Discussion

Periprosthetic femoral fractures arising decades after childhood tumor endoprosthesis implantation are uncommon and technically demanding. In pediatric osteosarcoma survivors, ongoing growth, asymmetric skeletal development, limb-length discrepancy, and secondary pelvic/spinal compensation can progressively alter load transfer, thereby predisposing to late failure modes such as loosening, osteolysis, wear, and periprosthetic fracture (10). In the present case, the distal knee tumor prosthesis remained well fixed, whereas proximal support had progressively deteriorated over time, leading to proximal migration and trochanteric involvement. The subsequent femoral neck fracture was likely multifactorial, related to stress concentration at a geometric discontinuity between the retained prosthesis, the augmentation construct, and native bone, compounded by disuse-related osteopenia and severe soft-tissue atrophy. Because the proximal femur lacked a usable medullary canal, a conventional femoral stem for THA was not feasible, and individualized reconstruction was required.

We adopted a component-preserving reconstruction using a customized monoblock stem combined with a dual-mobility head. Patient-specific planning based on CT/radiograph-derived three-dimensional reconstruction was performed to achieve a congruent prosthesis–prosthesis interface and to minimize intraoperative mismatch. Given the absence of a usable proximal canal and limited host bone stock, vancomycin-loaded PMMA was used to provide immediate fixation at the junction and to accommodate minor surface irregularities. To further enhance stability, multiple locking screws and a lateral anti-rotation plate were applied to resist torsional, shear, and axial loads, and predesigned holes facilitated soft-tissue (including trochanteric) reattachment. Dual-mobility articulation was selected to improve stability in the context of compromised abductor mechanics and complex revision anatomy.

A chronological summary of the key clinical events is provided in Table 1.

**Table 1:** Timeline of key clinical events.

Time	Event	Summary
2005 (age 8)	Initial surgery	Diagnosed with right distal femoral osteosarcoma and underwent tumor resection with knee tumor prosthesis replacement.
2018	Revision surgery	Revision surgery was performed at another hospital for proximal migration of the prosthesis and greater trochanteric perforation; detailed operative records were unavailable.
June 22, 2025	Injury	The patient sustained a fall and developed severe right hip pain with inability to bear weight.
June 25, 2025	Evaluation and planning	Radiographs and CT confirmed a displaced femoral neck fracture, while the distal prosthesis remained stable. A customized component-preserving reconstruction was planned.
July 16, 2025	Reconstruction	A customized monoblock stem was implanted and coupled to the retained distal prosthesis using PMMA cement, locking screws, and a lateral anti-rotation plate; a dual-mobility head was used.
Postoperative week 1	Early recovery	The patient began standing and walking exercises with rehabilitation guidance and was discharged.
3 months postoperatively	Follow-up	The patient could walk short distances without assistive devices; Harris Hip Score was 63.
Approximately 6 months postoperatively	Follow-up	The patient reported further functional improvement; Harris Hip Score improved to 81.

From a conceptual biomechanical perspective, load transfer in this reconstruction is expected to proceed from the customized monoblock stem to the socket region, across the cemented prosthesis-to-prosthesis interface, and then to the retained distal stem. This creates a noncontinuous load-sharing construct in which the junction may represent a mechanical weak point. Potential failure modes include interface debonding, fatigue failure at the prosthesis-cement-prosthesis junction, and progressive loosening under repeated torsional and shear loading. In this setting, auxiliary fixation with locking screws and a lateral anti-rotation plate was intended to enhance rotational control, resist shear forces, and reduce stress concentration at the junction.

Comparable reconstruction strategies have been reported when stable components can be retained. Corces et al. described a custom coupling device connecting proximal and distal femoral stems and noted heterogeneous complication profiles among coupling constructs (11). In an oncologic endoprosthesis setting, Wu et al. reported limited revision combined with massive allograft for periprosthetic femoral fractures around a tumor knee prosthesis, supporting reconstruction strategies that preserve stable components when feasible (12).

In the present case, total femoral replacement was not selected because the distal tumor prosthesis remained well fixed, and complete femoral replacement would have required a more extensive reconstruction with greater soft-tissue disruption and a more limited revision pathway if future failure occurred. In a young patient with high lifetime functional demands, preserving a stable distal component was considered a more reasonable first-line option.

Accordingly, we favored a component-preserving reconstruction strategy. This approach reduced the extent of surgical trauma and provided a component-preserving reconstruction strategy for the absent proximal femoral canal and complex revision anatomy. The customized socket-type prosthesis-to-prosthesis coupling design enabled linkage between the new proximal implant and the retained distal stem, while hybrid fixation with vancomycin-loaded PMMA cement, locking screws, and a lateral anti-rotation plate was intended to provide immediate mechanical stability. Nevertheless, this strategy has important limitations. Because the construct depends on a cement-bonded implant-cement-implant junction rather than a continuous metallic stem, potential risks include interface debonding, junctional fatigue failure, progressive loosening, and infection, which is broadly consistent with the complication patterns reported for coupling or connector constructs (11). Therefore, conclusions from this single case should be restricted to feasibility and early outcomes. The short-term prognosis appears encouraging, with the Harris Hip Score improving from 63 at 3 months to 81 at approximately 6 months; however, the long-term prognosis remains uncertain, and continued clinical and radiographic follow-up is warranted to monitor for junctional failure, loosening, infection, and progressive bone compromise.

At approximately 6 months, the patient reported ongoing functional improvement during telephone follow-up. Nonetheless, interpretation remains limited by the single-patient design, the lack of standardized functional outcome scores and biomechanical validation, and the inherent uncertainty of long-term durability in young survivors with high lifetime mechanical demands. In addition, because the 2018 revision was performed elsewhere and detailed operative documentation was unavailable, interpretation of the pre-existing reconstruction was based on available imaging, patient history, and intraoperative assessment. Future studies with larger cohorts and longer follow-up are needed to better define patient selection criteria, failure patterns, and durability of component-preserving reconstructions using customized monoblock stems in complex tumor prosthesis revisions.

## 4 Conclusion

This case demonstrates the feasibility of component-preserving reconstruction using a customized monoblock stem mated to a retained distal tumor prosthesis in the setting of an absent proximal femoral canal. Immediate postoperative radiographs showed satisfactory implant positioning, and early functional improvement was reported. Longer-term follow-up (preferably including imaging) and additional cases are needed to better define durability, complication profiles, and the generalizability of this component-preserving approach.

## Data Availability

All data generated or analyzed during this study are included in this article. Further inquiries can be directed to the corresponding author.
